# Metabolic studies in plant organs: don't forget dilution by growth

**DOI:** 10.3389/fpls.2014.00085

**Published:** 2014-03-11

**Authors:** Michel Génard, Valentina Baldazzi, Yves Gibon

**Affiliations:** ^1^UR 1115 Plantes et Systèmes de Culture Horticoles, Institut National de la Recherche AgronomiqueAvignon, France; ^2^UMR1332 Biologie du Fruit et Pathologie, Institut National de la Recherche AgronomiqueVillenave d'Ornon, France

**Keywords:** concentration, correlation, dilution, growth, metabolic, subcellular

## Introduction

The recent development of high-density technologies has enabled a dramatic extension of the exploration of living organisms. Such exploration typically consists in the parallel measurement of large numbers of compounds, at different developmental stages or following the application of contrasted environmental stimuli. Correlation analysis or more sophisticated statistical approaches are then used to identify clusters of co-varying compounds with the aim of reconstructing the underlying regulatory network governing system responses. Such approaches are now routinely used to identify predictive biomarkers (Steinfath et al., 2010; Riedelsheimer et al., 2012), including candidate genes (Carreno-Quintero et al., 2012).

Originally developed for unicellular organisms, these approaches are now applied to plants for the analysis of transcripts, proteins, metabolites (Gibon et al., 2006; Stitt et al., 2010; Liberman et al., 2012) and more recently enzymes activities (Gibon et al., 2004; Saito et al., 2008; Moreno-Risueno et al., 2010).

Most studies involve homogenization of specific tissues or even whole organs (e.g., fruit pericarp, leaf), without considering the subcellular localization of the measured compounds. Plant cells distinguish from other cells in possessing a large central vacuole, which size may vary dramatically between tissues, genotypes (species, cultivars) and developmental stages. Whereas young cells have small vacuoles, mature cells have large vacuoles that can encompass more than 95% of the cell volume.

Here, we will demonstrate that without taking into account the volumes of the cell compartments, the analyses of the dynamics of the compounds and the subsequent compound-compound correlation analyses might be biased, especially when the functional significance of the study is bound to concentrations, as it is the case for enzymes and metabolites. Possible correction strategies are discussed, with special emphasis to their pertinence and applicability to specific questions.

## Results

### Dilution by growth strongly affects metabolic concentrations

Let us consider for the sake of simplicity a cytosolic metabolite *M*. In many studies the metabolite concentration is expressed per volume (or, equivalently, per gram of fresh mass) of the whole tissue, as

[*Mt*] = *M*/*Vt* with *Vt* the volume of the tissue and *M* the metabolite quantity in this tissue. The metabolite being cytosolic, its concentration, to be physiologically meaningful, should be calculated as

[*Mc*] = *M*/*Vc* with *Vc* the volume of cytosol in the tissue.

**Figure 1:**
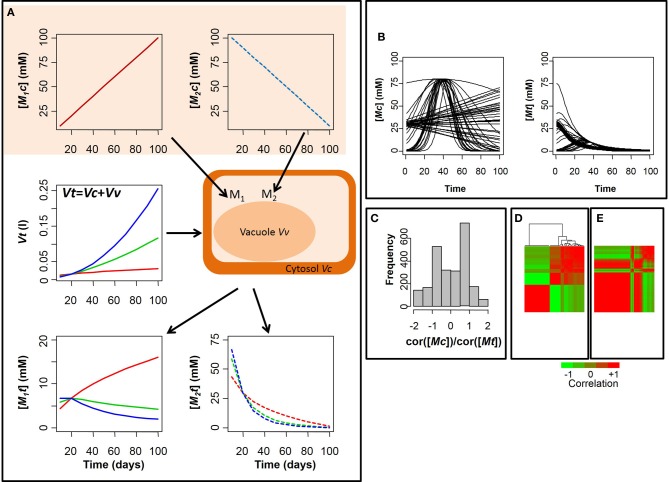
**Dynamics of the concentration of a metabolites in the cytosol and in the total tissue. (A)** Dynamics of the concentration of a metabolite *M* in the cytosol [*Mc*] and in the total tissue [*Mt*], assuming the volume of the cytosol *Vc* constant. Case of an increasing/decreasing cytosolic metabolite (respectively, M_1_ and M_2_) and three growth scenarii for *Vt* (red, green, and blue line); **(B)** Simulation of seasonal variation in the concentrations of 50 metabolites in cytosol [*Mc*] and total tissue [*Mt*], considering the blue scenario of **(A)** for the growth curve *Vt*; **(C)** distribution of the ratio metabolite- metabolite correlations in cytosol on metabolite-metabolite correlations in total tissue; heatmap of the metabolite-metabolite correlations and cluster analysis in cytosol **(D)** and total tissue **(E)**. The lines and column in **(E)** are ordered as in **(D)**. The values increase from the green to the red.

It is clear that [*Mt*] underestimates [*Mc*] because *Vt* is often much bigger than *Vc* due to the presence of other subcellular compartments (e.g., vacuole and plastids). More importantly, the ratio [*Mc*]/[*Mt*] = *Vt/Vc*, may not be constant among genotypes or during the development of organs where the ratio *Vt/Vc* strongly increases as the cell vacuole enlarges. This may lead to an erroneous interpretation of changes in metabolite concentration when comparing different experiments. Let's make an example. Assuming that *Vc* is constant and *Vt* increases with time, Figure 1A shows that depending on metabolite dynamics, the measured metabolite concentration [*Mt*] can be strongly different from the real cytosolic concentration, both quantitatively and qualitatively. In the extreme case, a decrease of [*Mt*] can be measured instead of an increase when the growth of the volume is faster than the metabolite accumulation in the cytosol (Figure 1A, blue line). This means that the seasonal variations of [*Mt*] during development of organs may not have any biological significance, reflecting the evolution of cell growth rather than a specific regulatory strategy.

Note that the effect of dilution depends on the specific features of both the growth curve and metabolite time course, so that it is difficult to correctly estimate the changes in [*Mc*] in absence of a good knowledge of the subcellular compartmentalization and its evolution in terms of relative volume occupancy within the cell. Of course the effect of dilution by growth is expected to be limited, at least in the last developmental stages, as long as vacuolar compounds are considered but, as shown before, it can become dramatic when compounds are localized in smaller organelles or in the cytosol.

### Metabolic correlation analysis are severely biased by dilution

The above considerations naturally extend to the analysis of correlations among concentrations of compounds, in a system-level perspective.

As an example, let's now consider 50 cytosolic metabolites, with linear or non-linear seasonal variation (Figure 1B). As shown in Figure 1B the pattern of correlation between the different metabolites can change dramatically due to dilution by growth. Indeed, most correlation values are altered and 47% of the metabolites display an opposite correlation when considering the total tissue in place of the cytosol (Figure 1C).

As a consequence, metabolites that could be meaningfully clustered together according to their cytosolic variations are now split into separate groups whereas non-correlated metabolites are incorrectly merged into a common cluster by dilution effect (Figures 1D,E). Overall, the effect of volume expansion is to increase positive correlation among cytosolic metabolites, damping variations at late developmental stages.

### Discussion and prospects

Plant development is a dynamic process that involves a complex series of molecular and biochemical events associated to volume expansion. Understanding its regulation is one of the great challenges of modern biology and has been the goal for many omics studies over the last years.

It is striking that in many studies fresh weight is used as the reference to express concentrations of biomolecules, irrespective of their subcellular localization. We show here that considering subcellular compartments is of primary importance for the correct interpretation of experimental results, especially when dealing with a system level perspective. Note that in the example we used, the organ volume was assumed to change with time but our conclusions are valuable for any other factor inducing organ volume variation, such as carbon or water stress, temperature, or genotypic traits. As a consequence, the bias induced by growth can potentially affect not only studies on dynamic profiling (Lombardo et al., 2011; Osorio et al., 2011, 2012) but also comparison among genotypes or the investigation of system response to stress (Cross et al., 2006; Sulpice et al., 2010; Osorio et al., 2012). For instance, Steinhauser et al. (2010) found that most of tomato enzyme activities decrease during fruit development and interpreted this as being at least in part due to vacuole expansion (in their study, the fruit volume varied from almost 0 to 60 cm^3^).

In the last few years a number of studies have pointed out subcellular compartmentalization as a key feature of plant cell biology and as an unavoidable requirement for accurate experimental measurements (Sun et al., 2004; Kruger et al., 2007; Fernie and Stitt, 2012) and models (Grafahrend-Belau et al., 2009; Hay and Schwender, 2011). In spite of these warnings, however, the impact of compartmentalization and especially of its variability among genotypes, experimental conditions or developmental stages is still largely underestimated. A key challenge for the future is therefore to improve existing methods to evaluate concentrations in cellular compartments. Recently, a first compartmentalized map of the metabolome of Arabidopsis has been proposed (Krueger et al., 2011) and the quantification of metabolite concentrations has been undertaken for specific organelles (Oikawa et al., 2011; Tohge et al., 2011). However, although many technical solutions ranging from sub-cellular fractionation (Gerhardt and Heldt, 1984) to *in situ* imaging (Okumoto et al., 2012) have been developed, none of them is yet fully applicable in routine (Krueger et al., 2012).

In the meanwhile, there are other ways to express concentrations of biomolecules that might prove more pertinent than fresh weight basis. The choice of the appropriate normalization factor depends on the scientific question of interest, the experimental protocol and the available data.

For tissues undergoing vacuolar expansion (Li et al., 2012), for instance, protein content represents an interesting option, as a proxy for the cytoplasmic compartment, assuming that proteins are by far less abundant in the vacuole. In many plants, a strong correlation has been showed between ploidy, nuclear size, and the volume of the cytoplasm (Sugimoto-Shirasu and Roberts, 2003), so that nuclear size could also be used as a proxy for the cytoplasmic compartment. However, methods for routine measurement of nuclear size have to be developed.

In absence of molecular measurements, dry mass, can be used as a normalization factor in situations where the water content and thus fresh mass can undergo strong variations (e.g., under water stress). If in addition to water, the content in storage compounds (starch, sucrose, amino acids, etc.) is expected to change, the above strategy can be iterated by expressing the concentration with respect to the structural dry mass (SDM) of the tissue, as:

$$\begin{array}{l} \left[X\right]=\left(\frac{DM}{FM}\right)\;\left(\frac{SDM}{DM}\right)\frac{M_{x}}{SDM}\text{ with}\\ \;SDM=DM\text{-}STO \end{array}$$

where *STO* is storage compounds content of the tissue. In this case, the first component describes the dilution due to water, the second describes the dilution due to the accumulation of storage carbohydrate and the third describes the concentration of *X*, independently of the dilution processes.

Another standardization used in water stress studies consists in expressing data on fresh mass at full turgor (Hummel et al., 2010). This method provides the possibility to evaluate the contribution of accumulated metabolites to osmotic adjustment. It nevertheless involves an additional and work intensive experimental step, which restricts its use to small scale experiments.

In the case of studies on leaf tissues, normalization with respect to leaf surface (Krapp et al., 1993) is generally advantageous from a practical point of view (collection of leaf discs of known surface) and perhaps also conceptually. In particular, various studies indicate that under stresses or genetic manipulations affecting photosynthesis, photosynthetic activity appears less sensitive when expressed on a mass-basis than on an area-basis, the latter being the one that correlates best with relative growth rate (Poorter et al., 2009). Chlorophyll content has also been used for leaves (Holaday et al., 1992) but its stability has to be checked.

Another way to circumvent the problem bound to subcellular volumes is to express data in a “semi-quantitative” way, i.e., not an absolute amount per unit of biomass but a relative amount. In the simplest case a signal obtained for a given analyte is divided by the signal obtained by another analyte, either internal, or external to the system under study. In the case of mass spectrometry-based metabolomics (Katajamaa and Oresic, 2007), reference samples are often used and metabolite levels are expressed as “fold-changes.” Their use assumes that variations in the relative sizes of compartments do not affect the output. However, only a fraction of the metabolites are solely located within one cell compartment, and their distributions between compartments may vary. The use of isotope-labeled biological standards does actually not solve this problem. Metabolomics also use single or multiple “internal” (added before the extraction) or “external” standards (added after the extraction). Metabolites are then expressed relative to one or more standards, but subcellular compartmentation is again critical. In contrast to metabolomics, transcriptomics (Irizarry et al., 2003; Bullard et al., 2010) and proteomics (Clough et al., 2012) usually involve normalization procedures where the whole population of analytes of a sample, an experiment or a series of experiments represents the reference, thus reducing the bias induced by changes in compartments/organelles volumes. Although such normalization probably is the most suitable for correlation analyses, it is considered as less robust for metabolites (Katajamaa and Oresic, 2007). It is worth mentioning that “semi-quantitative” data are suitable for studying fold-changes or for looking for correlations between variables, but not necessary for building mechanistic models (Rohwer, 2012). Indeed, whereas a range of integrative approaches can cope with alternatives to fresh weight to express amounts and/or activities of biomolecules, in others, such as mechanistic modeling, it will be crucial to evaluate the concentrations and thus the volume of the compartments where the enzymes or the metabolites are present. In that perspective, a challenging issue for the histologists moving to systems biology would be to find new techniques allowing quick measurement of compartment volumes and to feed existing databases on organelles dynamics (Mano et al., 2008, 2011).
